# Structurally engineered CNT-confined Mn_*x*_Ru_1−*x*_O_2_ catalysts for efficient acidic oxygen evolution at low Ru loading

**DOI:** 10.1039/d5sc04431f

**Published:** 2025-09-18

**Authors:** Xiaolin Zheng, Xiaofei Miao, Zijie Yang, Zhaoyan Luo, Jun Yu, Huiqi Li, Lei Zhang

**Affiliations:** a College of Chemistry and Environmental Engineering, Shenzhen University Shenzhen 518060 P.R. China huiqili@szu.edu.cn lei.zhang@szu.edu.cn; b College of Biomedical Engineering, Shenzhen University Shenzhen 518060 P.R. China

## Abstract

Developing acidic oxygen evolution reaction (OER) catalysts with low noble metal loading and high activity remains a critical challenge for advancing proton exchange membrane water electrolyzers. Herein, we report structurally engineered Mn_*x*_Ru_1−*x*_O_2_ catalysts confined on carbon nanotubes (CNTs), enabling highly dispersed active sites and remarkable catalytic activity at low Ru content. The uniform nanoscale coating of Mn_*x*_Ru_1−*x*_O_2_ along CNT sidewalls promotes Mn–O–Ru interfacial bonding and establishes an electron-bridge for enhanced charge transfer. The optimized CNT-(Mn_0.75_Ru_0.25_)O_2_ catalyst delivers a low overpotential of 120 mV at 10 mA cm^−2^ and an exceptional mass activity of 5549 A g_Ru_^−1^ at 270 mV—252 times that of commercial RuO_2_ (22 A g_Ru_^−1^). Combined X-ray spectroscopy, *in situ* Raman spectroscopy, and differential electrochemical mass spectrometry reveal that the electron-rich Ru centers stabilized by Mn–O bridges accelerate charge transfer and suppress Ru dissolution during the OER. Moreover, the CNT substrate and Ru incorporation synergistically generate abundant oxygen vacancies, significantly enhancing the catalytic activity through an improved lattice oxygen-mediated mechanism. This work highlights the critical role of CNT confinement and interfacial electronic modulation in decoupling noble metal usage from performance, offering a versatile design strategy for next-generation acidic OER catalysts.

## Abstract 2

Using structurally engineered Mn_*x*_Ru_1−*x*_O_2_ catalysts confined on CNTs, we clearly elucidate the electron modulation among CNTs, Mn, and Ru *via* Mn–O bridges. This enables excellent OER performance, with a mass activity of up to 5549 A g_Ru_^−1^ at 250 mV.

## Graphical abstract

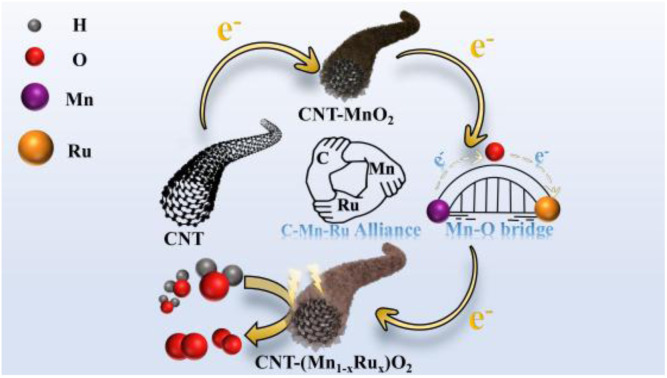

## Introduction

Proton exchange membrane water electrolyzers (PEMWEs) offer a promising route for sustainable hydrogen production due to their high current densities, rapid system response, and superior energy efficiency.^1–3^ However, the harsh oxidative and acidic environment on the anode side necessitates the use of noble metal-based oxygen evolution reaction (OER) catalysts, primarily Ru and Ir, which significantly increases system cost and limits large-scale deployment.^4–7^

Over the past decade, efforts to reduce noble metal usage in acidic OER catalysts have led to two competing trends: either maximizing intrinsic activity per noble metal atom (*e.g.*, *via* single-atom or heteroatom doping strategies), or lowering overpotential at the cost of increased loading. Yet, these approaches often encounter an inherent trade-off between activity and noble metal content. Few systems can simultaneously deliver overpotentials below 150 mV at 10 mA cm^−2^ and mass activities above 3000 A g_Ru_^−1^, as required for practical applications.^8–13^ In addition, many Ru-based materials undergo irreversible structural degradation due to the lattice oxygen-mediated mechanism (LOM), wherein lattice oxygen directly participates in O–O bond formation, accelerating metal dissolution and catalyst collapse.^14–16^

To overcome these limitations, a triple strategy is essential: (i) enhancing the atomic utilization of Ru *via* uniform dispersion, (ii) modulating the electronic structure to suppress the dissolution of active site Ru atoms, and (iii) improving catalyst activity through the introduction of abundant oxygen vacancies.^17–20^ Such an approach offers a promising pathway to break the traditional activity-cost-stability trade-off and enable the rational design of high performance and stable acidic OER catalysts.

Herein, we report structurally engineered Mn_*x*_Ru_1−*x*_O_2_ catalysts uniformly grown along carbon nanotube (CNT) sidewalls, forming a confined nanoscale interface that enables homogeneous Ru dispersion and C–Mn–O–Ru electronic coupling. Beyond physical confinement, CNTs play additional roles in modulating the catalyst structure and properties. During hydrothermal synthesis, CNTs react with KMnO_4_ to form chemically bonded MnO_2_ coatings, introducing oxygen vacancies and promoting uniform oxide dispersion. In addition, the CNTs enhance electrical conductivity, facilitate directional electron transfer from CNTs to Mn and then to Ru *via* Mn–O bridges, and mitigate structural degradation under acidic conditions.

These effects collectively contribute to the stabilization of Ru in a partially reduced state, suppressing over-oxidation and improving OER performance. As a result, the optimized CNT-confined (Mn_0.75_Ru_0.25_)O_2_ catalyst delivers a low overpotential of 120 mV at 10 mA cm^−2^ and a mass activity of 5549 A g_Ru_^−1^ at 270 mV, outperforming commercial RuO_2_ and the majority of reported Ru-based acidic OER catalysts. Comprehensive spectroscopic and electrochemical analyses reveal that the synergy of CNT confinement and interfacial electronic modulation is key to achieving high activity, low Ru loading, and enhanced stability. This work provides a viable pathway toward the design of cost-effective, durable acidic OER catalysts for scalable hydrogen production.

## Results and discussion

### Materials synthesis and characterization

**Scheme 1:**
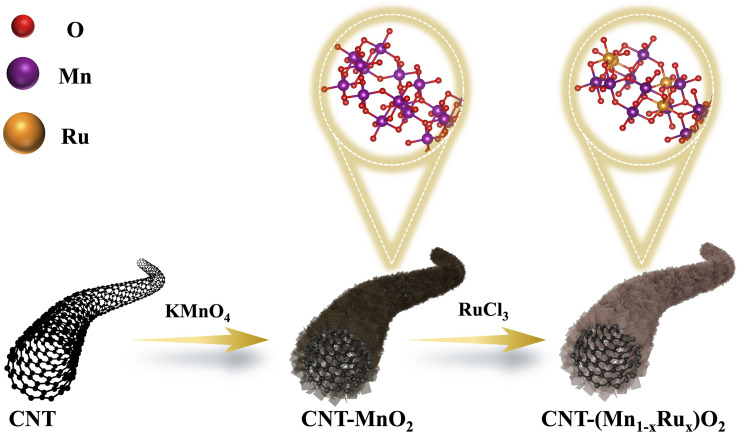
Schematic illustration of the synthesis of CNT-(Mn_1−*x*_Ru_*x*_)O_2_. KMnO_4_ oxidizes the CNT to form MnO_2_-coated CNT (CNT-MnO_2_), followed by RuCl_3_ treatment to generate Ru-substituted (Mn_1−*x*_Ru_*x*_)O_2_ domains confined on the CNT surface. The inset highlights the atomic-level incorporation of Ru into the MnO_2_ lattice, forming CNT-Mn–O–Ru bridges.

**Fig. 1:**
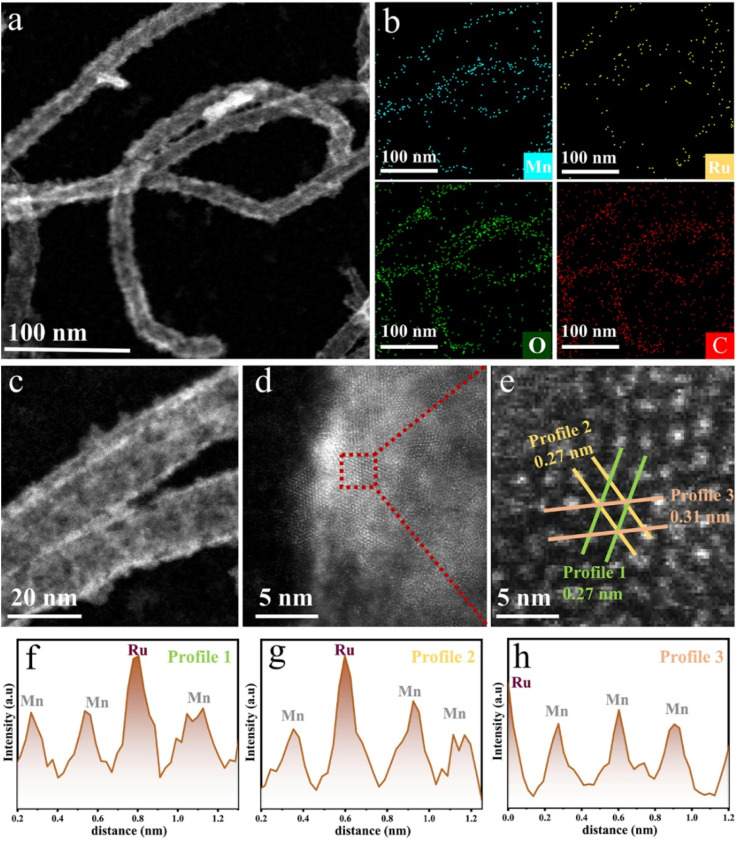
(a) HAADF-STEM image of CNT-(Mn_0.75_Ru_0.25_)O_2_. (b) EDS mapping of CNT-(Mn_0.75_Ru_0.25_)O_2_. (c) High-magnification STEM image showing the morphology from (a). (d) Atomic-resolution STEM image of a representative part selected from (c). (e) Enlarged view of the red-boxed region in (d). (f–h) The corresponding intensity profile of the line regions in (e).

To illustrate the synthesis strategy and structural evolution of our catalyst, a schematic diagram is shown in Scheme 1. Pristine carbon nanotubes (CNTs) were oxidized using KMnO_4_ to form a MnO_2_ shell, followed by RuCl_3_ treatment to introduce Ru and generate the (Mn_*x*_Ru_1−*x*_)O_2_ [*x* = 0.7–0.9, based on the inductively coupled plasma (ICP) result of Ru to Mn] alloy on the CNT surface (see Methods). To validate the proposed catalyst design, the crystalline structure of the catalysts was first analyzed using X-ray diffraction (XRD, Fig. S1). All samples exhibit a characteristic peak near 12.5°, characteristic of the tetragonal α-MnO_2_ phase (PDF#44-0141), distinguishing it from other MnO_2_ phases.^21^ Meanwhile, there were no detectable peaks associated with RuO_2_ and metallic Ru, indicating successful incorporation of Ru into the MnO_2_ lattice. Transmission electron microscopy (TEM) and high-angle annular dark-field scanning transmission electron microscopy (HAADF-STEM) were then employed to investigate the morphology and dispersion. As shown in Fig. 1a and S2, the oxide forms a continuous coating across the CNT surface, exhibiting nanoscale roughness and island-like surface features, with no apparent nanoparticle aggregation. This nanoscale confinement is critical for stabilizing the active phase and preventing particle growth, as observed in the previous studies.^14,18,22^ Elemental mapping (Fig. 1b) reveals a homogeneous distribution of Mn, Ru, and O across CNT surfaces, which further indicates the formation of an alloy rather than phase separation. High-resolution STEM images (Fig. 1c–e) exhibit distinct lattice fringes with spacings of 0.27 nm and 0.31 nm, corresponding to the (400) and (310) planes of α-MnO_2_, respectively. Moreover, intensity profiles (Fig. 1f–h and S3) show variations in atomic contrast consistent with Ru atoms substituting Mn within the oxide lattice. These structural features collectively indicate that Ru is incorporated into the MnO_2_ lattice, forming a homogeneous Mn_*x*_Ru_1−*x*_O_2_ solid solution.

**Fig. 2:**
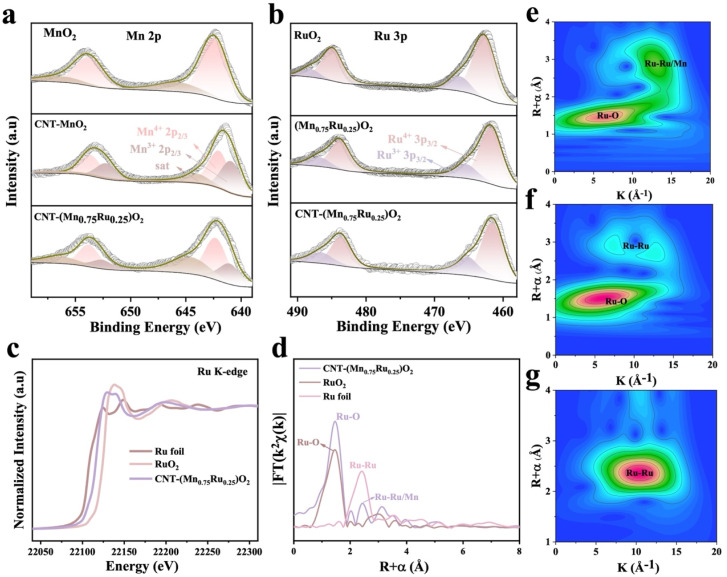
(a) Mn 2p XPS spectra of CNT-(Mn_0.75_Ru_0.25_)O_2_, CNT-MnO_2_ and MnO_2_. (b) Ru 3p XPS spectra of CNT-(Mn_0.75_Ru_0.25_)O_2_, (Mn_0.75_Ru_0.25_)O_2_ and RuO_2_. (c) Ru K-edge XANES spectra of CNT-(Mn_0.75_Ru_0.25_)O_2_, RuO_2_ and Ru foil. (d) The Fourier-transformed Ru K-edge EXAFS spectra of CNT-(Mn_0.75_Ru_0.25_)O_2_, RuO_2_ and Ru foil. (e–g) The wavelet transforms of the Ru K-edge EXAFS signals of CNT-(Mn_0.75_Ru_0.25_)O_2_, RuO_2_ and Ru foil.

To further understand the electronic structure and coordination environment of CNT-(Mn_*x*_Ru_1−*x*_)O_2_ catalysts, X-ray photoelectron spectroscopy (XPS) and X-ray absorption spectroscopy (XAS) analyses were conducted.^23–25^ The Mn 2p_3/2_ peak of CNT-MnO_2_ exhibits a 0.4 eV negative shift relative to that of MnO_2_, indicating that the introduction of CNTs promotes electron transfer from CNTs to Mn. For CNT-(Mn_0.75_Ru_0.25_)O_2_, the Mn 2p_3/2_ peak shows a 0.24 eV positive shift compared to CNT-MnO_2_, suggesting that Ru doping increases the oxidation state of Mn, leading to electron loss by Mn (Fig. 2a). When comparing (Mn_0.75_Ru_0.25_)O_2_ with commercial RuO_2_, the Ru 3p_3/2_ peak of (Mn_0.75_Ru_0.25_)O_2_ undergoes a 0.4 eV negative shift, indicating that Ru is in a partially reduced state. The opposite shifts between Mn and Ru confirm the existence of electron coupling through the oxygen bridge, with electrons transferring from Mn to Ru. Additionally, the Ru 3p_3/2_ peak of CNT-(Mn_0.75_Ru_0.25_)O_2_ shows a 0.2 eV negative shift compared to (Mn_0.75_Ru_0.25_)O_2_ (Fig. 2b). Taken together, these shifts confirm that in CNT-(Mn_0.75_Ru_0.25_)O_2_, electrons transfer from C to Mn and then to Ru through the Mn–O bridge. In addition, the progressive increase in work function across CNTs, MnO_2_, (Mn_*x*_Ru_1−*x*_)O_2_, and RuO_2_ enables spontaneous electron transfer along this sequence, which is consistent with the observed XPS peak shifts and supports the proposed C to Mn to Ru charge redistribution.^26–28^ This charge redistribution stabilizes Ru in a partially reduced state, which is expected to mitigate over-oxidation and dissolution under acidic OER conditions (Tables S1 and S2).^29^ This effect also can effectively optimize the electronic structure of active sites, thereby modulating both activity and stability.^30–32^

**Fig. 3:**
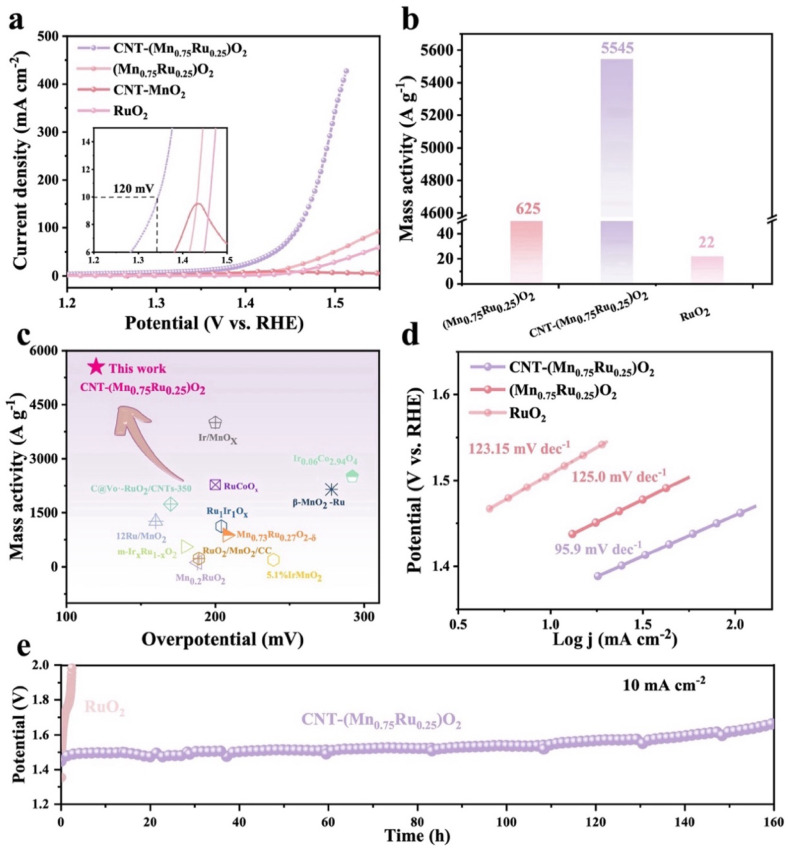
(a) OER performance of the catalysts in 0.1 M HClO_4_ solution. (b) Mass activity at 1.5 V of the catalysts. (c) Comparison of OER overpotential and mass activity of previously reported electrocatalysts. (d) Tafel slope of the catalysts. (e) Durability test of CNT-(Mn_0.75_Ru_0.25_)O_2_ and RuO_2_.

The O K-edge soft X-ray absorption near-edge structure (XANES) spectrum of CNT-(Mn_0.75_Ru_0.25_)O_2_ (Fig. S4) exhibits a noticeable decrease in the intensity of the Ru–O hybridization peak and a negative energy shift relative to CNT-MnO_2_, indicating an increased electron density localized on oxygen atoms. Meanwhile, the incorporation of Ru into CNT-MnO_2_ increases the density of unoccupied states and lowers the *t*_2g_/*e*_g_ ratio, which is known to enhance the adsorption of oxygenated intermediates. This electronic structure is consistent with the partial reduction of Ru species observed from XPS results.^33,34^ The Ru K-edge XANES spectra (Fig. 2c) further support this conclusion. A distinct negative shift in the absorption edge of CNT-(Mn_0.75_Ru_0.25_)O_2_ relative to commercial RuO_2_was observed, indicating a lower Ru oxidation state. Moreover, the corresponding white line intensity is also reduced, indicating a lower density of unoccupied Ru 4d states and weaker Ru–O hybridization. Fourier-transformed Ru K-edge EXAFS spectra (Fig. 3d, S5, S6 and Table S3) display coordination peaks at ∼1.44 Å (Ru–O), ∼2.42 Å (Ru–Ru/Mn), and ∼2.39 Å (Ru–Ru). As revealed by Ru K-edge EXAFS fitting results, the Ru–O bond length in CNT-(Mn_0.75_Ru_0.25_)O_2_ (1.95 Å) is slightly shorter than that in RuO_2_ (1.96 Å). This slight shortening can be rationalized by anisotropic Coulomb interactions induced by Mn incorporation into the Ru local environment, which has been reported as a structural signature of solid-solution formation in Ru–Mn oxides.^53^ Based on the comprehensive analysis above, the CNT-(Mn_*x*_Ru_1−*x*_)O_2_ catalyst is consistent with a solid solution structure. And the corresponding coordination number of Ru–O bonds in CNT-(Mn_0.75_Ru_0.25_)O_2_ (2.9) is lower than that in RuO_2_ (4.6) and Ru foil (4.6), indicating that the catalyst contains abundant oxygen vacancies. Wavelet transform (WT) analysis (Fig. 2e–g) further validates the coexistence of Ru–O, Ru–Mn, and Ru–Ru coordination environments.^13^ These XAS results and XPS analyses collectively confirm that the Ru sites in CNT-(Mn_0.75_Ru_0.25_)O_2_ are in an electron-rich state, which originates from CNT-to-Mn-to-Ru electron transfer and is stabilized by Mn–O bridges.

### Electrocatalytic activity

The electrocatalytic performance of CNT-(Mn_*x*_Ru_1−*x*_)O_2_ catalysts was systematically evaluated for the acidic oxygen evolution reaction (OER) in the O_2_-saturated 0.1 M HClO_4_ electrolyte, using a standard three-electrode configuration. Linear sweep voltammetry (LSV) measurements were conducted with carbon paper (1 × 1 cm^2^) as the working electrode, a platinum wire as the counter electrode, and a reversible hydrogen electrode (RHE) as the reference electrode. All potentials were corrected for *iR* compensation to ensure accurate comparison. As shown in Fig. 3a and S7, CNT-(Mn_0.75_Ru_0.25_)O_2_ exhibits the lowest overpotential of 120 mV at 10 mA cm^−2^ and 270 mV at 400 mA cm^−2^, significantly outperforming both the CNT-free (Mn_0.75_Ru_0.25_)O_2_ (200 mV at 10 mA cm^−2^) and commercial RuO_2_ benchmark (230 mV at 10 mA cm^−2^). This enhancement demonstrates the critical role of the CNT support in dispersing the active sites and facilitating synergistic interactions between Mn and Ru. The inductively coupled plasma-optical emission spectrometry (ICP-OES) analysis confirms the low Ru content in the CNT-supported catalysts, with Ru loadings of 1.7 wt%, 3.2 wt%, 3.9 wt%, and 4.9 wt% for CNT-(Mn_0.9_Ru_0.1_)O_2_, CNT-(Mn_0.8_Ru_0.2_)O_2_, CNT-(Mn_0.75_Ru_0.25_)O_2_, and CNT-(Mn_0.7_Ru_0.3_)O_2_, respectively (Table S4). Notably, despite the low Ru content, CNT-(Mn_0.75_Ru_0.25_)O_2_ achieves a remarkable mass activity of 5549 A g_Ru_^−1^ at 1.5 V *vs.* RHE, which is ∼252 times higher than that of commercial RuO_2_ (22 A g_Ru_^−1^) and substantially higher than that of most reported Ru-based catalysts (Fig. 3b, c, S8 and Table S5). Although the Ru content in CNT-(Mn_0.75_Ru_0.25_)O_2_ is 3.9 wt%, the corresponding Ru loading on the carbon paper working electrode is only 0.077 mg cm^−2^. At this low loading, the CNT-(Mn_0.75_Ru_0.25_)O_2_ delivers an overpotential of 120 mV at 10 mA cm^−2^, along with outstanding mass activity (Table S6). This result highlights that the strategic incorporation of Ru into the MnO_2_ matrix, mediated by the CNT support, enables efficient atomic dispersion of Ru and optimizes electronic interactions, thereby achieving exceptional catalytic performance at minimal noble metal content.

Kinetic analysis based on Tafel slopes further reveals that CNT-(Mn_0.75_Ru_0.25_)O_2_ exhibits the lowest slope of 95.9 mV dec^−1^, significantly lower than those of commercial RuO_2_ (123.1 mV dec^−1^) and (Mn_0.75_Ru_0.25_)O_2_ (125.0 mV dec^−1^, Fig. 3d and S9).^35^ This indicates that the CNT-Mn-Ru system effectively facilitates the reaction kinetics by modulating the electronic structure and lowering the energy barrier for the OER process. Electrochemical impedance spectroscopy (EIS) measurements (Fig. S10) reveal that CNT-(Mn_0.75_Ru_0.25_)O_2_ possesses the lowest charge transfer resistance (*R*_ct_) among all tested samples, further confirming the role of CNTs in providing a highly conductive network that accelerates electron transport.^36–38^ The electrochemical surface area (ECSA), determined from the dependence of double-layer capacitance (*C*_dl_) on different scan rates (5 mV s^−1^ to 25 mV s^−1^), further supports the enhanced activity of CNT-(Mn_0.75_Ru_0.25_)O_2_. CNT-(Mn_0.75_Ru_0.25_)O_2_ exhibits an ECSA of 159.9 mF cm^−2^, which is 7.5 and 10.5 times higher than that of (Mn_0.75_Ru_0.25_)O_2_ (21.3 mF cm^−2^) and commercial RuO_2_ (15.2 mF cm^−2^), respectively (Fig. S11 and S12).^34,39^ This substantial increase reveals the dual role of CNTs: promoting high-density dispersion of active sites and maximizing their electrochemical accessibility. Long-term durability tests at a constant current density (10 mA cm^−2^, Fig. 3e) demonstrate that CNT-(Mn_0.75_Ru_0.25_)O_2_ retains stable operation for over 160 hours. To evaluate catalyst degradation, ICP-OES analysis of the post-electrolysis electrolyte revealed that only 4.9% of Ru was dissolved, substantially lower than the ∼19% reported for commercial RuO_2_ under similar acidic conditions, indicating enhanced resistance to Ru dissolution under acidic OER conditions.^40^ In contrast, commercial RuO_2_ suffers from rapid degradation, with significant activity loss observed within 3 hours. Meanwhile, CV tests at different scan rates were performed on the post-stability sample. The *C*_dl_ (Fig. S13) decreased by approximately 26% compared with the sample before the stability test. Combined with ICP and HAADF-STEM analysis, the decrease in double-layer capacitance supports the conclusion that catalysts agglomeration occurred during the stability test. To further understand the origin of performance decay after 160 h, TEM and HAADF-STEM images (Fig. S14) were recorded which showed that the homogeneous Mn_*x*_Ru_1−*x*_O_2_ solid solution structure is maintained. The initially uniform distribution became more aggregated on the CNT surface, indicating particle growth. XRD analysis (Fig. S15) reveals sharpened diffraction peaks, confirming increased crystallinity. This aggregation likely reduces the accessible active surface area, contributing to the observed decline in OER performance. Nonetheless, the well-dispersed (Mn_*x*_Ru_1−*x*_)O_2_ architecture before operation plays a key role in achieving high activity at low Ru content and future optimization will focus on mitigating particle growth to enhance durability.

### Catalytic mechanism

To unravel the origin of the enhanced OER, we first examined the oxygen species in CNT-(Mn_0.75_Ru_0.25_)O_2_, (Mn_0.75_Ru_0.25_)O_2,_ CNT-MnO_2_ and MnO_2_ catalysts by XPS. As shown in Fig. S16a, the O 1s spectra of all catalysts can be deconvoluted into three components corresponding to lattice oxygen (O_lat_), oxygen vacancies (O_v_), and adsorbed oxygen species (O_ads_). Notably, the O_v_ percentage of CNT-(Mn_0.75_Ru_0.25_)O_2_ (60.4%) is significantly larger than that of other catalysts, indicating a higher concentration of oxygen vacancies in the CNT-Mn-Ru system. And the percentages of CNT-MnO_2_ (28.8%) and (Mn_0.75_Ru_0.25_)O_2_ (38.2%) are both larger than that of MnO_2_ (18.8%), indicating that CNTs and Ru can promote the generation of more oxygen vacancies (Fig. S16b). These results suggest that both the incorporation of Ru in MnO_2_ and the CNT support facilitates the generation of oxygen vacancies. The observed increase in O_v_ concentration can be rationalized by the following mechanism. Oxygen vacancies in metal oxides can be introduced through cation doping or anion substitution. Incorporation of cations with lower oxidation states or lower oxygen vacancy formation energies tends to promote the formation of oxygen vacancies *via* a charge compensation mechanism. In this work, the introduction of low-valent Ru^3+^ into the MnO_2_ lattice induces such a compensation process, thereby increasing the concentration of oxygen vacancies.^41^ This defect engineering not only enhances the intrinsic activity by providing additional active sites for O–O coupling, but also modulates the surface electronic structure to favor intermediate adsorption during the OER.^42,43^

**Fig. 4:**
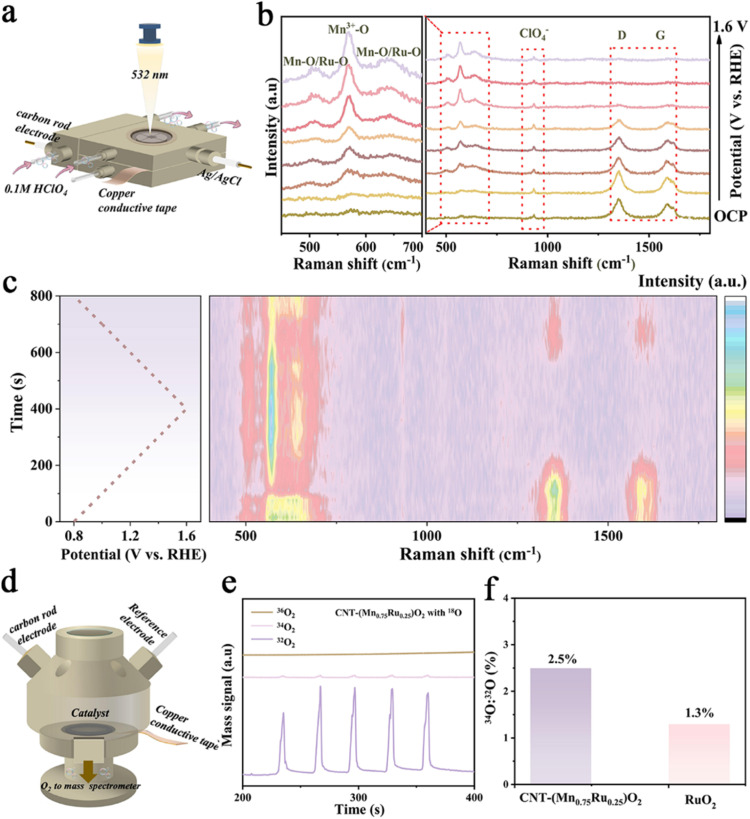
(a) Schematic of the *in situ* Raman spectroscopy setup. (b) *In situ* Raman spectra of CNT-(Mn_0.75_Ru0_.25_)O_2_ collected at increasing potentials from OCP to 1.6 V *vs.* RHE. (c) Corresponding Raman contour plots of CNT-(Mn_0.75_Ru_0.25_)O_2_ during CV cycling from 0.8–1.6 V *vs.* RHE at 1 mV s^−1^. (d) Schematic of the DEMS setup. (e) Mass spectrometry traces of ^32^O_2_, ^34^O_2_, and ^36^O_2_ signals during potential cycling of CNT-(Mn_0.75_Ru_0.25_)O_2_ in H_2_^16^O–HClO_4_ electrolyte. (f) Comparison of the ^34^O_2_/^32^O_2_ peak area ratio of CNT-(Mn_0.75_Ru_0.25_)O_2_ and RuO_2_.

The dynamic structural evolution of the catalyst under OER conditions was further investigated by *in situ* Raman spectroscopy. Since the water band remains constant with increasing potential, all Raman spectra were normalized to the intensity of the water peak to minimize the effects of bubble interference and enable accurate comparison of intermediate-related features. As illustrated in Fig. 4a, an H-type flow cell was used to monitor the real-time structural evolution of catalysts under potential control. The vibrational spectra of RuO_2_ are observed in Fig. S17, showing characteristic Ru–O stretching modes.^44^ For CNT-(Mn_0.75_Ru_0.25_)O_2_, the Raman peaks at 510 cm^−1^ and 637 cm^−1^ are assigned to Mn–O/Ru–O stretching vibrations, while the peak at ∼580 cm^−1^ corresponds to the Mn^3+^–O band, consistent with previous reports.^25,45–48^ Additionally, carbon-related features are observed at ∼1350 cm^−1^ (D band), ∼1580 cm^−1^ (G band), and ∼2700 cm^−1^ (2 G band),^49,50^ corresponding to defect-induced vibrations, in-plane sp^2^ carbon stretching, and the second-order overtone, respectively. Notably, the intensities of Mn–O/Ru–O and Mn^3+^–O vibrational modes increase markedly from 0.8 V to 1.6 V *vs.* RHE, reflecting the potential-dependent accumulation of lattice oxygen intermediates (Fig. 4b). In addition, increasing peak intensity with the applied voltage suggests that the oxygen vacancies can participate in the progress of the reaction, whereas CNT-MnO_2_ shows negligible spectral variation over the same potential range (Fig. S18a). The corresponding contour plots (Fig. 4c and S18b) demonstrate reversible modulation of the characteristic peaks through CV cycling, indicating dynamic reconstruction of reversible oxygen vacancy changes of the CNT-(Mn_0.75_Ru_0.25_)O_2_ during the OER, which does not occur in CNT-MnO_2_. Notably, the CNT-(Mn_0.75_Ru_0.25_)O_2_ catalyst exhibits no significant red or blue shift of the Ru–O vibrational band at increasing potential, in contrast to the blue shift observed in CNT-MnO_2_. This spectral invariance suggests that the formation of higher-valence Ru species is effectively inhibited, likely due to directional electron transfer mediated by Mn–O bridges. These bridges facilitate electron transfer from CNTs to Ru centers, mitigating over-oxidation under acidic OER conditions and thereby enhancing catalyst durability.^52^ These observations reveal that the Mn–O–Ru interaction plays a key role in enabling dynamic surface restriction under OER conditions, which promotes the reversible binding of intermediates. Simultaneously, the CNT provides robust structural support, ensuring the stable dispersion of active sites throughout the reaction.^51^

To further probe the oxygen evolution pathway, we performed *in situ* differential electrochemical mass spectrometry (DEMS) measurements using ^18^O-labeled catalysts CNT-(Mn_0.75_Ru_0.25_)O_2_ and commercial RuO_2_ (Fig. 4d). Prior to DEMS analysis, the catalysts were pre-labelled by CV cycling in 0.1 M HClO_4_ prepared with H_2_^18^O. After the labelling process, the evolved O_2_ during the OER was monitored in a 0.1 M HClO_4_ electrolyte prepared with H_2_^16^O, allowing us to distinguish lattice oxygen (^18^O) and oxygen derived from water (^16^O). The evolution of ^36^O_2_ (^18^O^18^O), ^34^O_2_ (^16^O^18^O) and ^32^O_2_ (^16^O^16^O) was quantitatively tracked to probe the oxygen evolution mechanism of catalysts.^52,53^ As shown in Fig. 4e and S19, during the OER process of both catalysts, only ^34^O_2_ and ^32^O_2_ were detected, with no ^36^O_2_ observed, confirming that both catalysts follow a lattice oxygen-mediated mechanism (LOM). The ratio of ^34^O_2_ to ^32^O_2_ (Fig. 4f) for CNT-(Mn_0.75_Ru_0.25_)O_2_ was determined to be 2.5%, higher than that of commercial RuO_2_ (1.3%), indicating greater lattice oxygen participation in the CNT-(Mn_0.75_Ru_0.25_)O_2_. This phenomenon is attributed to the abundant oxygen vacancies on the CNT-(Mn_0.75_Ru_0.25_)O_2_, which can be labelled by ^18^O and then reversibly participate in the OER process. This trend aligns well with the higher oxygen vacancy concentration derived from XPS O 1s deconvolution and the potential-dependent reversibility observed in *in situ* Raman spectroscopy, suggesting that the CNT-confined Mn–O–Ru framework stabilizes Ru sites in a partially reduced state and inhibits over-oxidation.^54,55^

**Fig. 5:**
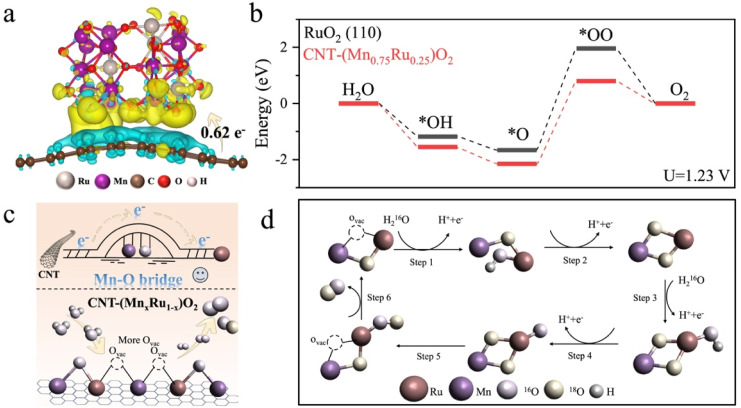
(a) Charge distribution analysis of CNT-(Mn_0.75_Ru_0.25_)O_2_. Yellow region indicates charge accumulation, while the blue region indicates charge reduction. (b) The calculated reaction pathways of CNT-(Mn_0.75_Ru_0.25_)O_2_ and RuO_2_(110) for acidic OER at *U* = 1.23 V. Schematic illustration of the LOM pathway during acidic OER and (c and d) enhanced LOM pathway in CNT-(Mn_*x*_Ru_1−*x*_)O_2_ with improved oxygen vacancy dynamics and structural stability.

To further explore the reaction mechanism, density functional theory (DFT) calculations were performed (Fig. 5a, b and S20).^58^Fig. 5a presents the differential charge analysis results of CNT-(Mn_0.75_Ru_0.25_)O_2_ with Bader charge calculations showing that 0.62*e*− transfer from CNTs to (Mn_0.75_Ru0_.25_)O_2_. As shown in Fig. 5b, the rate-determining step (RDS) for both CNT-(Mn_0.75_Ru_0.25_)O_2_ and RuO_2_ involves the formation of the *OO intermediate. The energy barrier for the RDS of CNT-(Mn_0.75_Ru_0.25_)O_2_ is lower than that of RuO_2_ (110), indicating an improvement in the reaction kinetics. Combined with the experimental evidence from DFT calculations, it is shown that there exist more favorable oxygen evolution pathways between CNT-(Mn_0.75_Ru_0.25_)O_2_ and commercial RuO_2_. We believe that the classic LOM in RuO_2_ involves direct participation of lattice oxygen in the O–O formation process. However, the highly symmetric rutile structure of RuO_2_, while intrinsically active, renders it susceptible to excessive lattice oxygen activation under acidic OER conditions, promoting the formation of unstable RuO_4_^2−^ intermediates (Fig. S21).^56,57^ This over-oxidation pathway inevitably accelerates Ru dissolution and compromises catalyst stability. In contrast, as shown in Fig. 5c and d, the CNT-(Mn_*x*_Ru_1−*x*_)O_2_ adopts a structurally and electronically distinct configuration that enables a more favorable LOM process.^58^ The incorporation of Mn and the CNT support induces a highly dispersed architecture in which electron-rich Ru centers are stabilized by Mn–O bridges and abundant oxygen vacancies. These features synergistically enhance both activity and stability. On the one hand, the Mn–O bridges facilitate directional electron transfer from CNTs to Ru during the OER, serving as a protective pathway that mitigates over-oxidation of the Ru under acidic conditions.^28^ On the other hand, *in situ* Raman spectroscopy corroborated that CNT-(Mn_0.75_Ru_0.25_)O_2_ exhibits reversible, potential-dependent modulation of the Mn–O/Ru–O and Mn^3+^–O vibrational bands, while RuO_2_ shows limited dynamic flexibility.^53^ The enhanced and reversible Raman signals in CNT-(Mn_*x*_Ru_1−*x*_)O_2_ suggest that oxygen vacancies not only provide additional adsorption sites for intermediates but also enable structural adaptability during OER cycling, facilitating efficient formation and desorption of *O species.^59–61^

Taken together, these results establish a coherent mechanistic framework: the synergistic combination of the CNT support, Mn incorporation, and Ru dispersion in CNT-(Mn_*x*_Ru_1−*x*_)O_2_ creates a unique Mn–O-bridge network that enables directional electron transfer, modulates surface electronic states, and tunes oxygen vacancy concentration. This CNT-confined Mn–O–Ru framework effectively improves the usage of Ru atoms, mitigates Ru over-oxidation and dissolution, and promotes a more stable and efficient OER process under acidic conditions. In contrast, RuO_2_ suffers from its symmetric crystal structure and strong lattice oxygen participation, which ultimately accelerates Ru leaching and deactivation.^62,63^

## Conclusions

In summary, we report structurally engineered CNT-confined (Mn_*x*_Ru_1−*x*_)O_2_ catalysts that achieve outstanding acidic OER performance at low Ru loading. The uniform nanoscale coating of (Mn_*x*_Ru_1−*x*_)O_2_ along the CNT sidewalls not only promotes highly dispersed active sites and abundant oxygen vacancies, but also facilitates directional electron transfer through Mn–O bridges. This unique electronic architecture stabilizes Ru in a partially reduced state, effectively mitigating over-oxidation and dissolution pathways that typically plague Ru-based catalysts under acidic conditions. Combined spectroscopic and electrochemical analyses reveal that tuning the local electronic environment and oxygen vacancy concentration is essential for suppressing dissolution of Ru, thereby balancing catalytic activity and stability. This work highlights the importance of CNT confinement and Mn–O bridge-mediated electronic coupling in modulating Ru reactivity and durability. Overall, our findings establish a versatile design strategy for developing robust, high-performance acidic OER catalysts that decouple noble metal usage from activity, offering a promising pathway for advancing PEMWE technologies.

## Author contributions

L. Z., H. L. and X. Z. conceived the research and wrote the paper. X. Z. and X. M. performed catalysts synthesis and general characterization. Z. Y. performed DEMS, supervised by Z. L. J. Y. assisted X. Z. in the XAS measurements. L. Z. and H. L. supervised the research. All the authors contributed to the discussions and revisions of the manuscript.

## Conflicts of interest

The authors declare no competing interests.

## Supplementary Material

SC-OLF-D5SC04431F-s001

## Data Availability

The datasets are available from the corresponding author on reasonable request. Data for this article, including experimental precedure, supplementary tables and figures, characterization data of the products, *etc.*, are available in the SI. See DOI: https://doi.org/10.1039/d5sc04431f.
