# Supplementary material for: Structurally engineered CNT-confined MnxRu1−xO2 catalysts for efficient acidic oxygen evolution at low Ru loading
Source: Chem Sci. 2025 Sep 18;16(42):19820–9. doi: 10.1039/d5sc04431f (PMC12462428; doi:10.1039/d5sc04431f)
Supplement: SC-016-D5SC04431F-s001 [file SC-016-D5SC04431F-s001.pdf]

Supplementary Information (SI) for Chemical Science.
This journal is © The Royal Society of Chemistry 2025

Supporting Information

Structurally Engineered CNT-Confined $Mn_xRu_{1-x}O_2$ Catalysts for Efficient Acidic Oxygen Evolution at Low Ru Loading

Xiaolin Zheng,[a†] Xiaofei Miao,[ab†] Zijie Yang [a], Zhaoyan Luo [a], Jun Yu [a], Huiqi Li, *[a] and Lei Zhang *[a]

[a] College of Chemistry and Environmental Engineering, Shenzhen University, Shenzhen, 518060, P.R. China
[b] College of Biomedical Engineering, Shenzhen University, Shenzhen, 518060, P.R. China
Corresponding e-mail: huiqili@szu.edu.cn; lei.zhang@szu.edu.cn

Materials and Methods

Chemicals

All chemicals, including potassium permanganate ($KMnO_4$, ≥99.5%, Shanghai Lingfeng Chemical Reagent Co., Ltd), ruthenium trichloride ($RuCl_3$, 45-55%, Aladdin), carbon nanotubes tube (CNT, 99%, Shenzhen Suiheng Technology Co., Ltd.), ruthenium(IV) oxide (RuO_2, 99.9%, Macklin), perchloric acid ($HClO_4$, 70%~72%, Aladdin), $H_2{}^{18}O$ (≥97 atom% ^{18}O, Aladdin), absolute alcohol (98%, Aladdin), and millipore water (0.07 μS cm^{-1}), were used without further purification.

Synthesis of CNT-MnO_2

Firstly, 30 mg of CNT was dispersed in 50 ml of millipore water by sonication for 30 min. Then 0.48 mmol of $KMnO_4$ was added dropwise to the CNT, vigorous stirring at room temperature for 1 hour. The mixture was transferred to an autoclave and heated at 120 °C for 12 hours. Upon cooling to room temperature, the resulting black floccule were washed five times with millipore water to obtain CNT-MnO_2.

Synthesis of CNT-$(Mn_xRu_{1-x})O_2$

Deionized CNT-MnO_2 in 90 ml millipore water and inject 0.05 mmol, 0.1 mmol, 0.16 mmol, 0.2 mmol $RuCl_3$, vigorous stirring at room temperature for 12 hours. Washed five times, and then calcined in air at 80 °C for 2 h and at 200 °C for 1 h at an elevated temperature program of 0.5 °C min^{-1} to obtain CNT-$(Mn_{0.9}Ru_{0.1})O_2$, CNT-$(Mn_{0.8}Ru_{0.2})O_2$, CNT-$(Mn_{0.75}Ru_{0.25})O_2$, and CNT-$(Mn_{0.7}Ru_{0.3})O_2$.

Characterizations

The crystal structures of CNT-MnO_2 CNT-$(Mn_{0.9}Ru_{0.1})O_2$, CNT-$(Mn_{0.8}Ru_{0.2})O_2$, CNT-$(Mn_{0.75}Ru_{0.25})O_2$, CNT-$(Mn_{0.7}Ru_{0.3})O_2$ were investigated by X-ray diffraction (XRD) (PANalytical, Empyrean). The size, morphology, and d-spacing of the samples were characterized by transmission electron microscopy (TEM), High-angle annular dark-field scanning transmission electron microscopy (HAADF-STEM), and combined with energy-dispersive X-ray spectroscopy (EDX). The samples' chemical state was analyzed by X-ray photoelectron spectroscopy (XPS) and X-ray absorption spectroscopy (XAS). The composition of the catalyst was determined by inductively

coupled plasma atomic emission spectrometer (ICP-OES) on Thermo Fisher iCAP PRO. Raman spectra were tested on Renishaw inVia with an excitation laser of 532 nm.

Electrochemical measurements in three-electrode cell

Electrochemical performances of the catalysts were evaluated in a 0.1 M $HClO_4$ electrolyte with three-electrode cell connected to the electrochemical workstation biology. Reversible hydrogen electrode (RHE) and Pt as the reference and counter electrode. 0.5 mg catalysts were dispersed in a mixture of 980 μl of deionized water and isopropanol with a volume ratio of 1:1 with 20 μl of 10% nafion solution as binder. For the OER experiment, CV tests were performed rate of 10 mV s^{-1} from 0.8 V to 1.65 V in order to activate catalyst, LSV with a scanning rate of 5 mV s^{-1} was measured in the potential rang of 1.2-1.65 V vs. RHE.

Differential electrochemical mass spectroscopy (DEMS) measurements

DEMS experiments were conducted using a standard three-electrode electrochemical cell, with a saturated Ag/AgCl electrode as the reference and a platinum wire as the counter electrode. All measurements were conducted in a 0.1 M $HClO_4$ electrolyte. Isotopic labeling of the catalyst was performed by running 10 cycles CV at a scan rate of 5 mV s^{-1} in 0.1 M $HClO_4$ prepared with $H_2{}^{18}O$, within a potential range of 1.2 to 1.65 V vs. RHE. This procedure enabled the incorporation of ^{18}O into the lattice oxygen prior to DEMS analysis.

After the labeling step, the ^{18}O -labeld electrode was thoroughly rinsed 10 times with $H_2{}^{16}O$ to eliminate any residual $H_2{}^{18}O$ in the catalyst layer or electrolyte film. Subsequently, DEMS measurements were conducted in regular $H_2{}^{16}O$-based 0.1 M $HClO_4$ electrolyte. The evolution of O_2 was monitored by tracking the ion current signals corresponding to m/z = 32 ($^{16}O_2$), 34 ($^{16}O^{18}O$), and 36 ($^{18}O_2$). The relative intensities of these signals were used to evaluate the participation of lattice oxygen in OER.

Following this, the ^{18}O-labeled electrode was washed 10 times with ^{16}O water to eliminate any remaining ^{18}O-enriched water.

Ultimately, linear sweep voltammetry (LSV) cycles within the aforementioned potential window and at the same scan rate were applied to the ^{18}O-labeled electrode in a $HClO_4$ solution prepared with ^{16}O water. Concurrently, mass spectrometry was used for real-time detection of the gaseous products generated during the oxygen evolution reaction (OER).

Calculation method

We performed the relevant computational work based on density functional theory (DFT)[1-2]. The calculations were implemented in the Vienna Ab initio Simulation Package (VASP), employing the technical scheme that includes the projector augmented-wave (PAW) method[3-4] and the plane-wave basis set. For the treatment of exchange-correlation potential, the generalized gradient approximation (GGA) was selected in combination with the Perdew-Burke-Ernzerhof (PBE) parametrization scheme[5]. To eliminate the influence of interactions between adjacent images, a vacuum layer with a thickness of approximately 15 Å was specifically set. The energy cutoff in the calculations was determined to be 450 eV, and the van der Waals (vdW) correction (DFT-D3) mechanism was introduced simultaneously. The integration operation of the Brillouin zone achieved sampling through a 2×2×1 Monkhorst-Pack grid. The structures were subjected to sufficient relaxation treatment until the maximum force exerted on each atom dropped below 0.05 eV/Å, and the energy convergence accuracy was controlled at the level of 10^{-5} eV. The bottom atoms of the surface slab remained fixed at their bulk positions.

Calculation of the mass activity

The mass activity ($j_{mass\ activity}$ (A g^{-1})) of the CNT-(Mn_xRu_{1-x})O_2 and RuO_2 catalysts was determined using equation：

$$j_{mass\ activity} = \frac{i \times S}{m}$$

i (mA cm^{-2}) is current density; S (cm^2) is geometric area; m (mg) is the calculated Ru mass loaded onto glassy carbon based on the results of ICP-OES analysis.

Calculation of the specific current density per electrochemically active surface area

The electrochemically active surface area (ECSA) for all electrocatalysts was estimated from the electrochemical double-layer capacitance (C_{dl}) of the catalytic surface according to equation

$$ECSA = \frac{C_{dl}}{C_s}$$

where C_{dl} is measured from the scan-rate-dependent CVs in the non-Faradaic region of 0.9–1.0 V versus RHE in 0.1 M $HClO_4$ with the scan rate of 5, 10, 15, 20, and 25 mV s^{-1}. The Cs is 0.035 mF cm^{-2}.

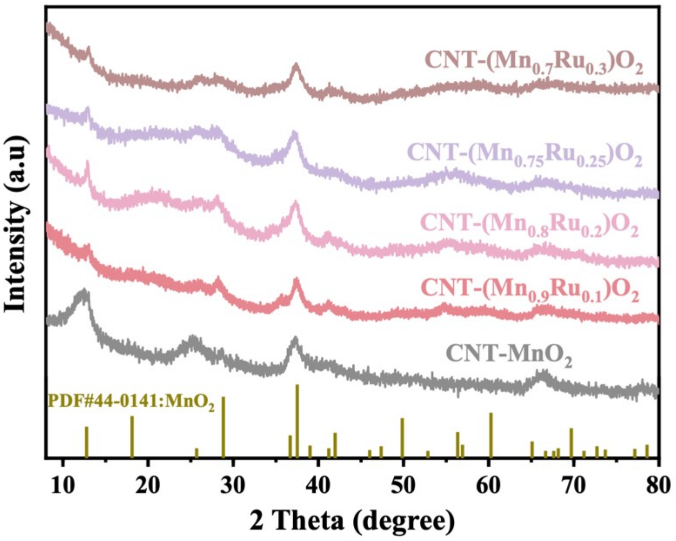

Figure S1. XRD patterns of CNT-MnO_2, CNT-$(Mn_{0.9}Ru_{0.1})O_2$, CNT-$(Mn_{0.8}Ru_{0.2})O_2$, CNT-$(Mn_{0.75}Ru_{0.25})O_2$, and CNT-$(Mn_{0.7}Ru_{0.3})O_2$.

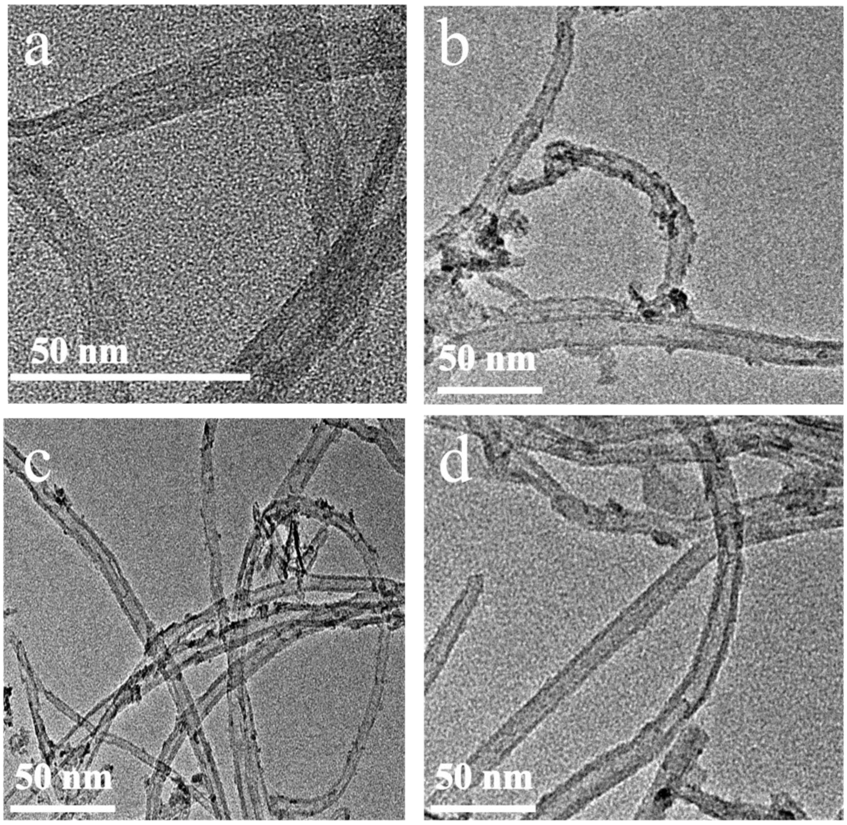

Figure S2. TEM images of (a) CNT-MnO_2, (b) CNT-$(Mn_{0.9}Ru_{0.1})O_2$, (c) CNT-$(Mn_{0.8}Ru_{0.2})O_2$, and (d) CNT-$(Mn_{0.7}Ru_{0.3})O_2$.

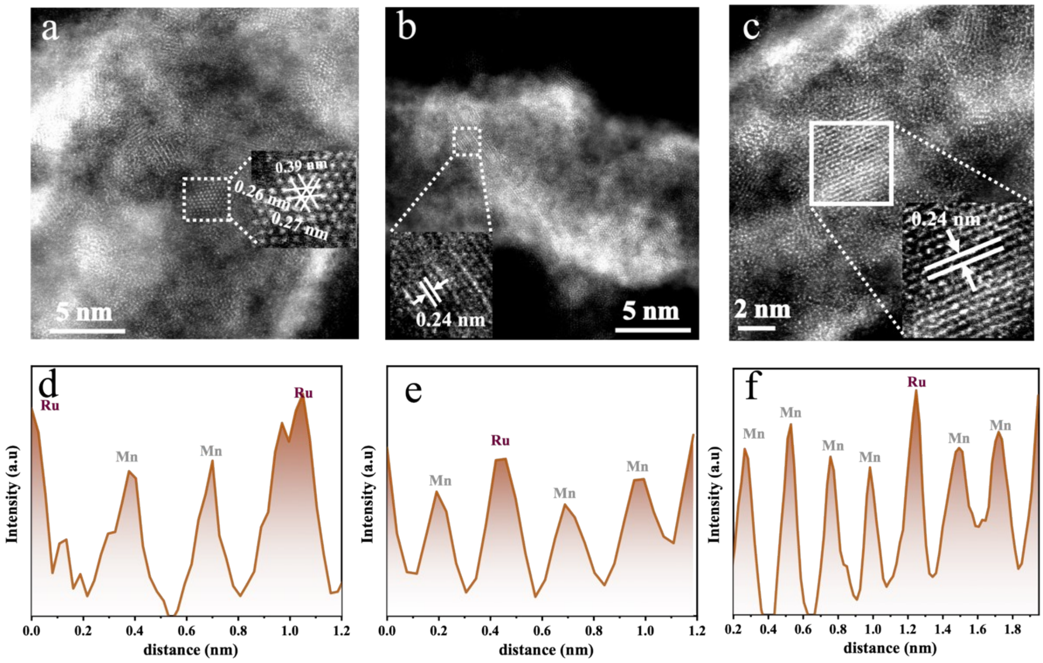

Figure S3. (a, b, and c) HAADF-STEM image of CNT-$(Mn_{0.75}Ru_{0.25})O_2$ and magnification of the individual particles. (d, e, and f) The corresponding intensity of the line regions in (a, b, and c).

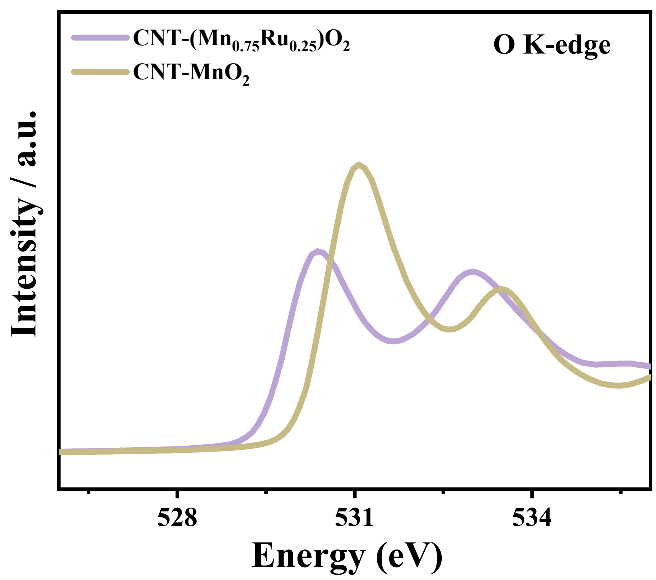

Figure S4. Normalized O K-edge XAS spectra for CNT-$(Mn_{0.75}Ru_{0.25})O_2$ and CNT-MnO_2.

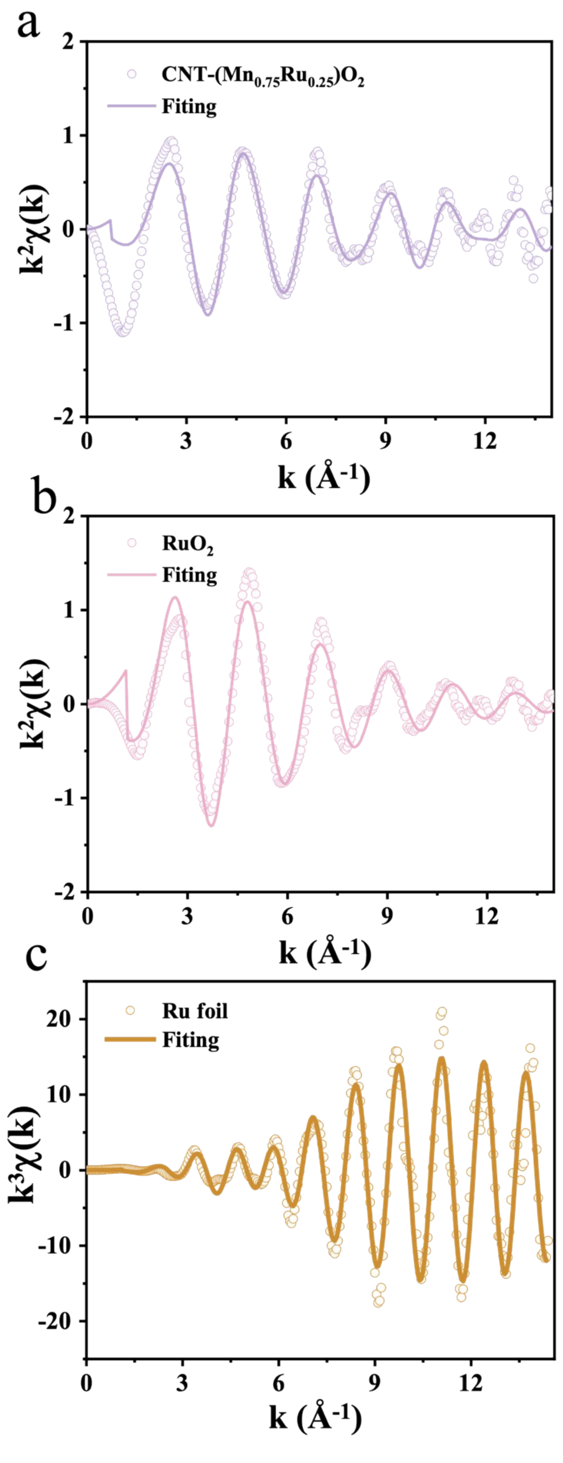

Figure S5. EXAFS fitting results of (a) CNT-($Mn_{0.75}Ru_{0.25}$)O_2, (b) RuO_2, and (c) Ru foil.

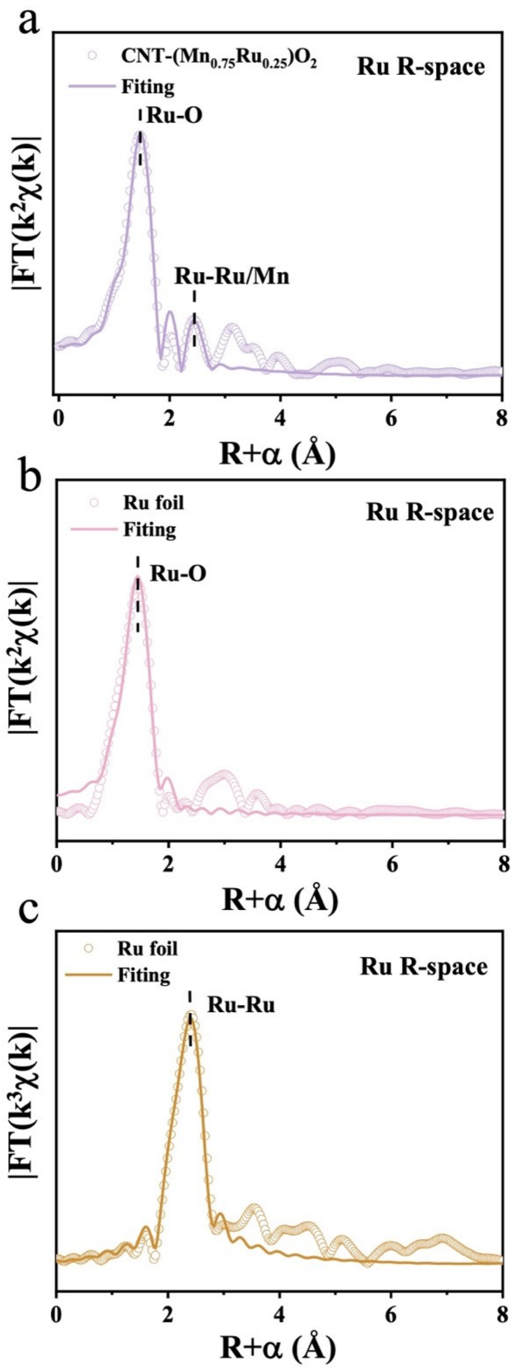

Figure S6. The Fourier-transformed magnitude fitting result of (a) CNT-$(Mn_{0.75}Ru_{0.25})O_2$, (b) RuO_2, and (c) Ru foil.

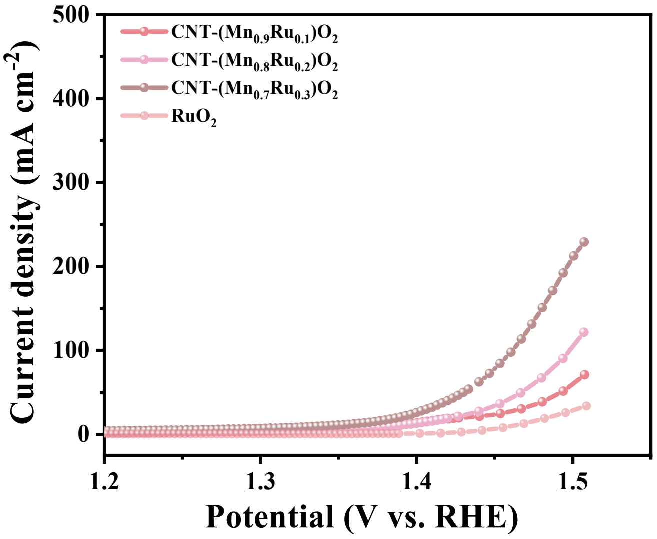

Figure S7. OER performance of CNT-($Mn_{0.9}Ru_{0.1}$)O_2, CNT-($Mn_{0.8}Ru_{0.2}$)O_2, CNT-($Mn_{0.7}Ru_{0.3}$)O_2, and RuO_2 catalysts in 0.1 M $HClO_4$ solution.

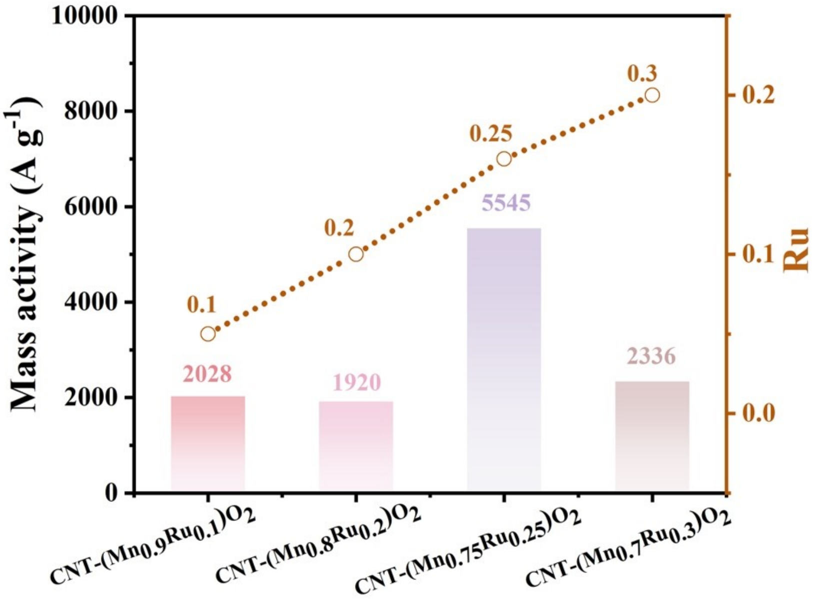

Figure S8. Mass activity at 1.5 V of CNT-$(Mn_{0.9}Ru_{0.1})O_2$, CNT-$(Mn_{0.8}Ru_{0.2})O_2$, CNT-$(Mn_{0.75}Ru_{0.25})O_2$, and CNT-$(Mn_{0.7}Ru_{0.3})O_2$ catalysts.

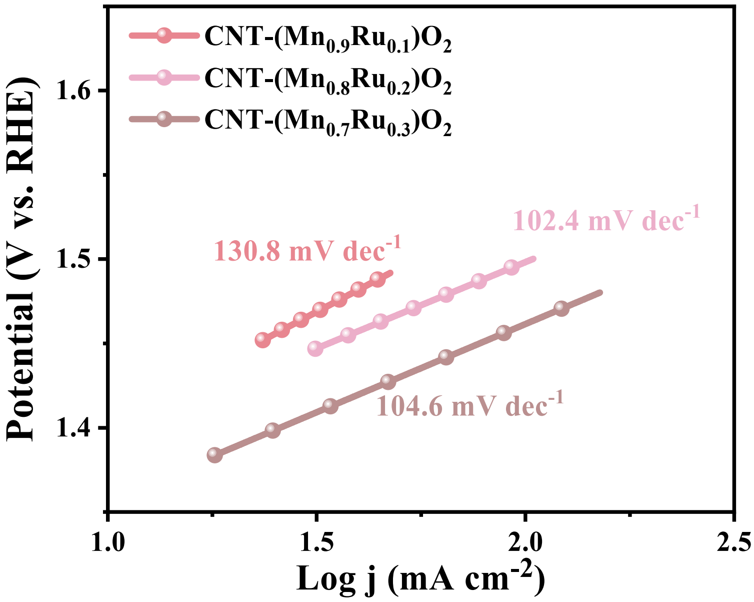

Figure S9. Tafel slop of CNT-($Mn_{0.9}Ru_{0.1}$)O_2, CNT-($Mn_{0.8}Ru_{0.2}$)O_2, and CNT-($Mn_{0.7}Ru_{0.3}$)O_2 catalysts.

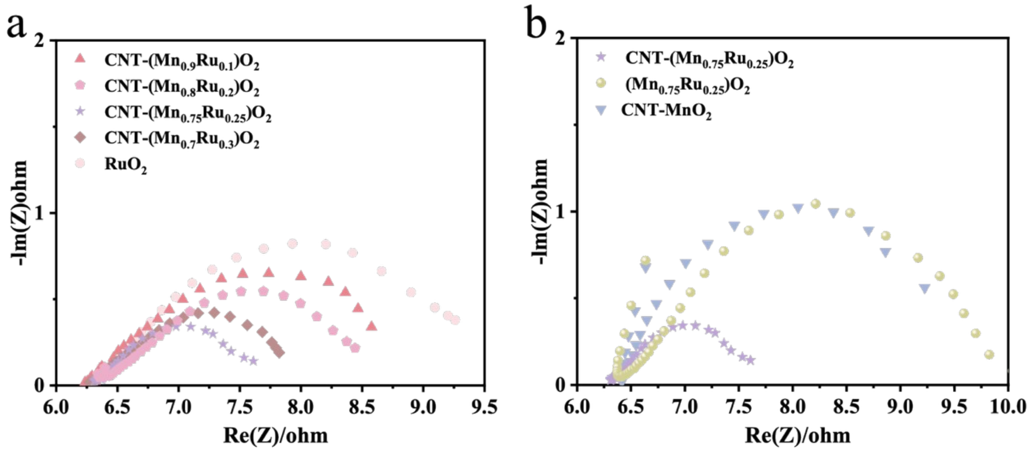

Figure S10. EIS spectra of (a) CNT-$(Mn_xRu_{1-x})O_2$ and RuO_2, (b) CNT-$(Mn_{0.75}Ru_{0.25})O_2$, $(Mn_{0.75}Ru_{0.25})O_2$, and CNT-MnO_2.

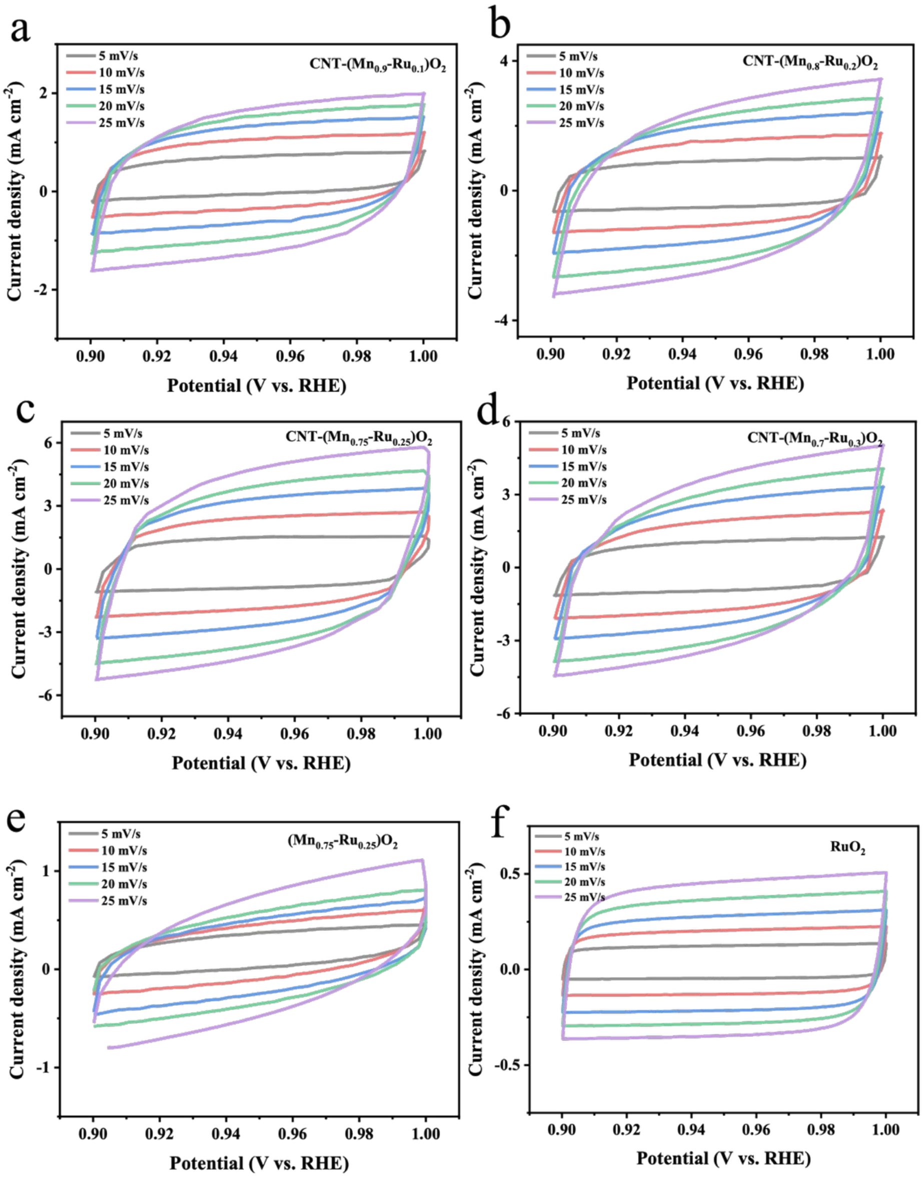

Figure S11. CV curves of (a) CNT-($Mn_{0.9}Ru_{0.1}$)O_2, (b) CNT-($Mn_{0.8}Ru_{0.2}$)O_2, (c) CNT-($Mn_{0.75}Ru_{0.25}$)O_2, (d) CNT-($Mn_{0.7}Ru_{0.3}$)O_2, (e) ($Mn_{0.75}Ru_{0.25}$)O_2, and (f) RuO_2 in the range of 0.9 to 1.0 V vs. RHE, with the scan rates increasing from 5 mV to 25 mV s^{-1}.

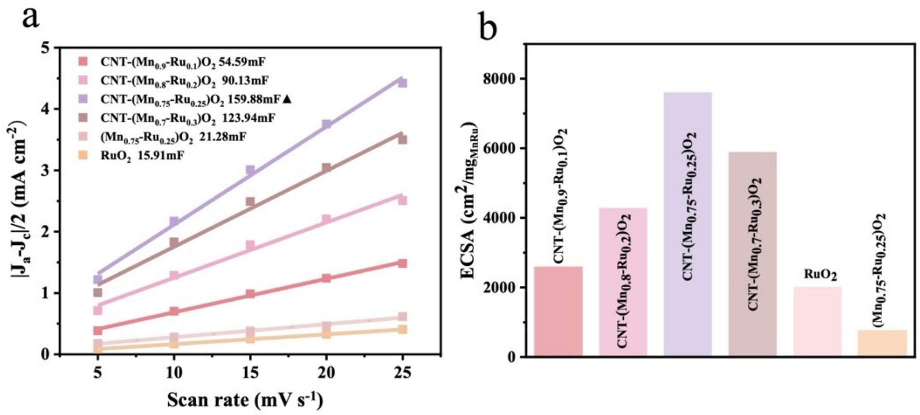

Figure S12. (a) The calculate C_{dl} and (b) ECSA of CNT-($Mn_{0.9}Ru_{0.1}$)O_2, CNT-($Mn_{0.8}Ru_{0.2}$)O_2, CNT-($Mn_{0.75}Ru_{0.25}$)O_2, CNT-($Mn_{0.7}Ru_{0.3}$)O_2, RuO_2, and ($Mn_{0.75}Ru_{0.25}$)O_2.

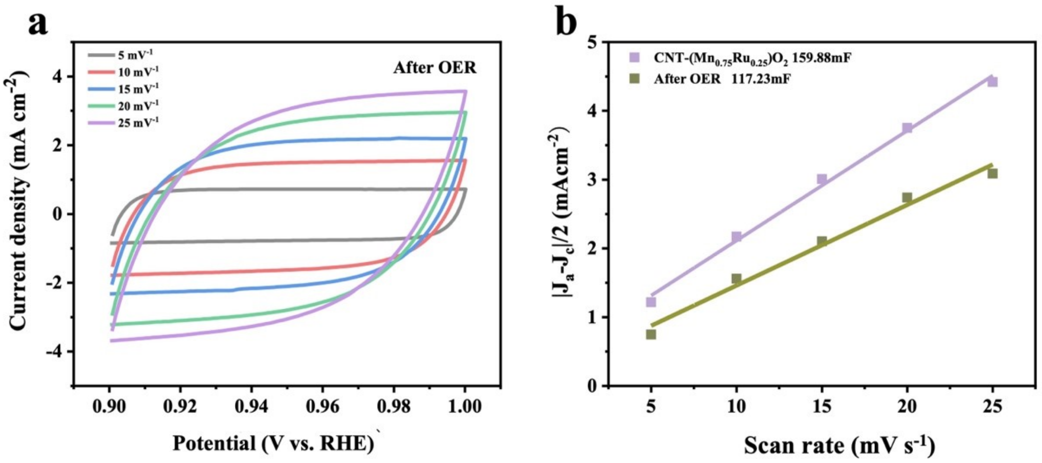

Figure S13. (a) CV curves of after stability test CNT-$(Mn_{0.75}Ru_{0.25})O_2$ and (b) The calculate C_{dl} of CNT-$(Mn_{0.75}Ru_{0.25})O_2$ after stability test.

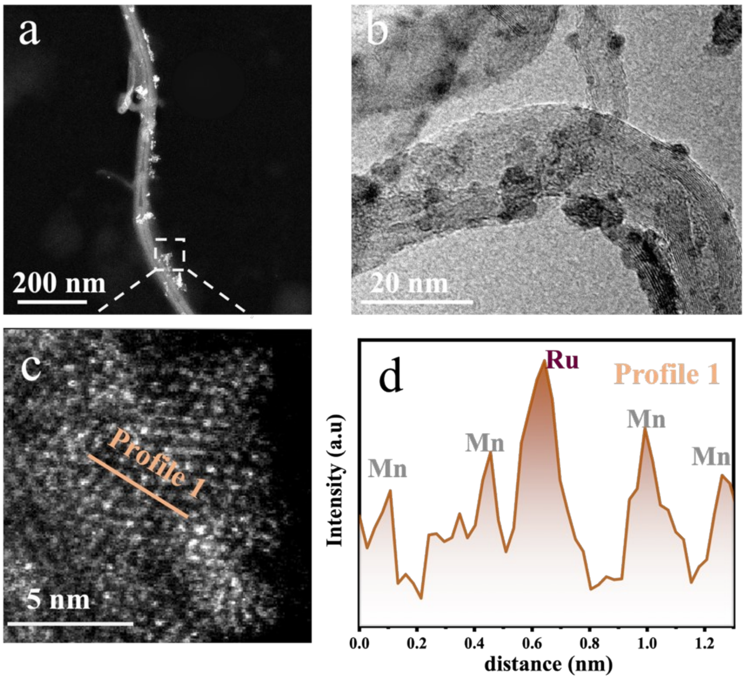

Figure S14 Characterizations of CNT-$(Mn_{0.75}Ru_{0.25})O_2$ after stability test. (a) HAADF-STEM , (b)TEM, (c) high-magnification HAADF-STEM view of the selected region in (a), (d) intensity of the profile along the marked line in (c).

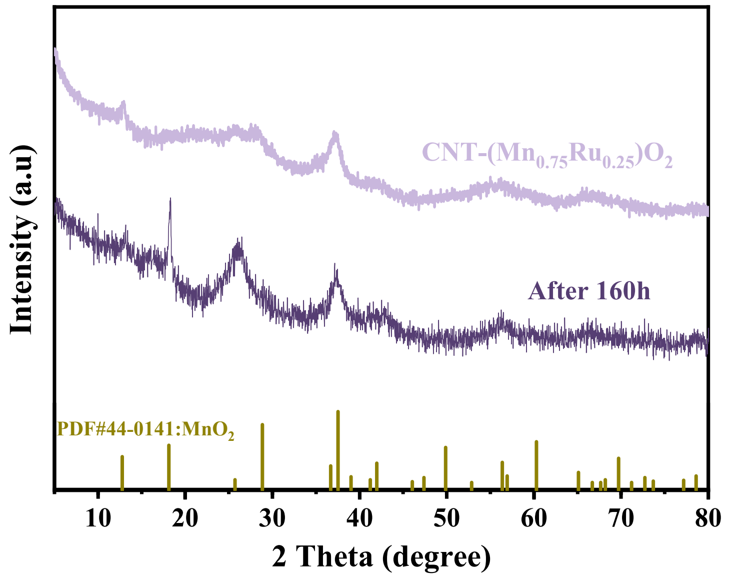

Figure S15 XRD patterns of CNT-$(Mn_{0.75}Ru_{0.25})O_2$ before and after stability test.

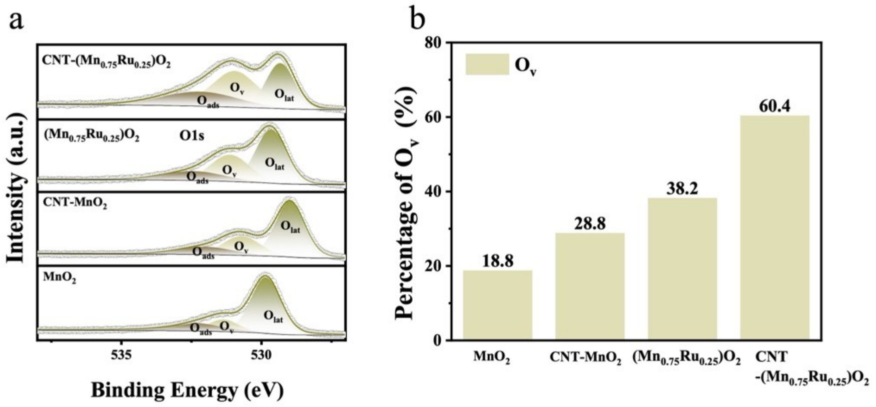

Figure S16. (a) O 1s XPS spectra and (b) O_v percentage of MnO_2, CNT-MnO_2, $(Mn_{0.75}Ru_{0.25})O_2$, and CNT-$(Mn_{0.75}Ru_{0.25})O_2$.

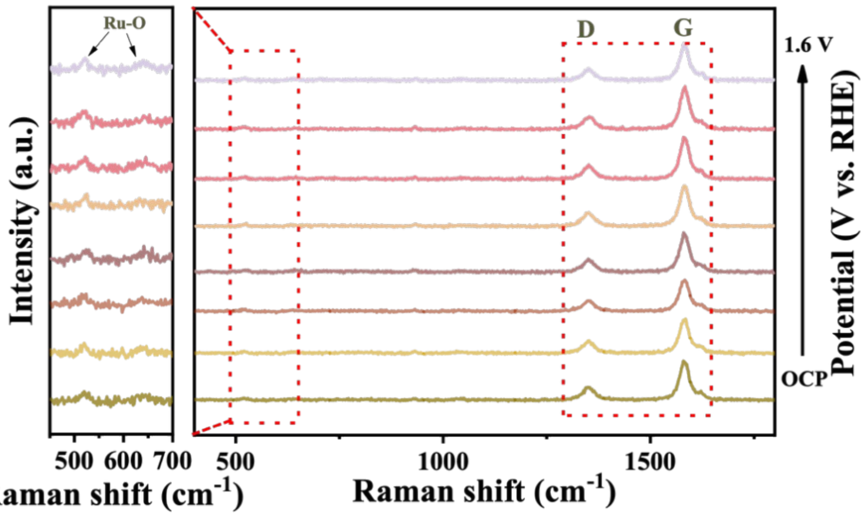

Figure S17. *In-situ* Raman spectra of RuO_2 obtained under various applied potentials.

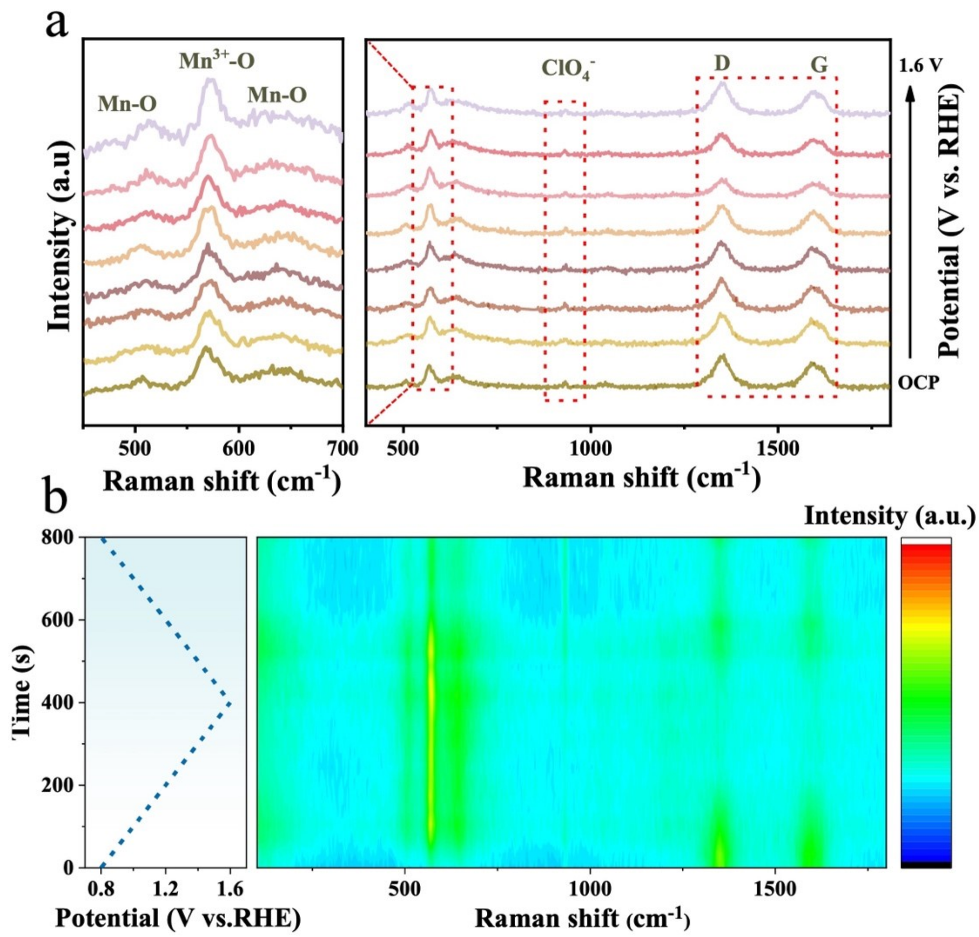

Figure S18. (a) *In-situ* Raman spectra of CNT-MnO_2 obtained under various applied potentials. (b) In situ Raman spectra of CNT-MnO_2 obtained under CV cycling between 0.8 V and 1.6 V vs. RHE at 1 mV s^{-1}.

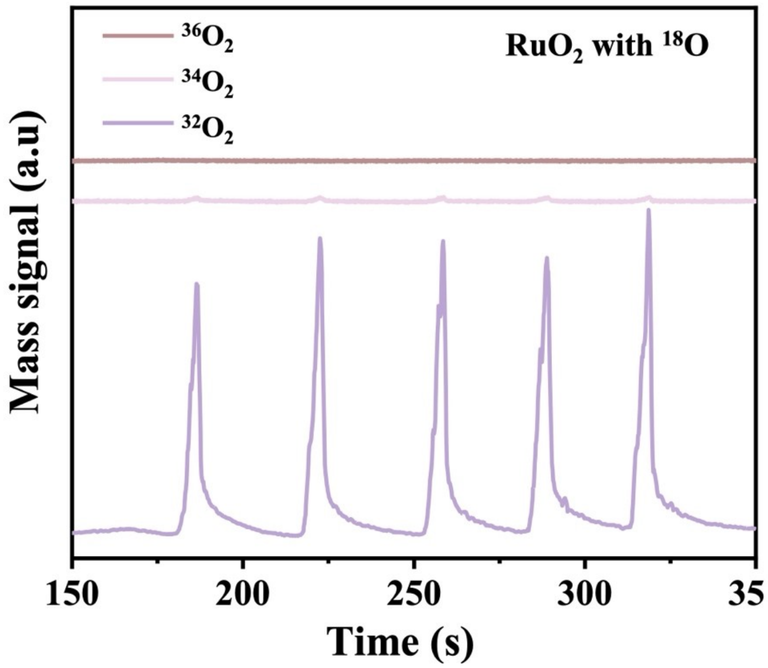

Figure S19. Mass spectrometry traces of $^{32}O_2$, $^{34}O_2$, and $^{36}O_2$ signals during potential cycling of RuO_2 in $H_2{}^{16}O$-$HClO_4$ electrolyte.

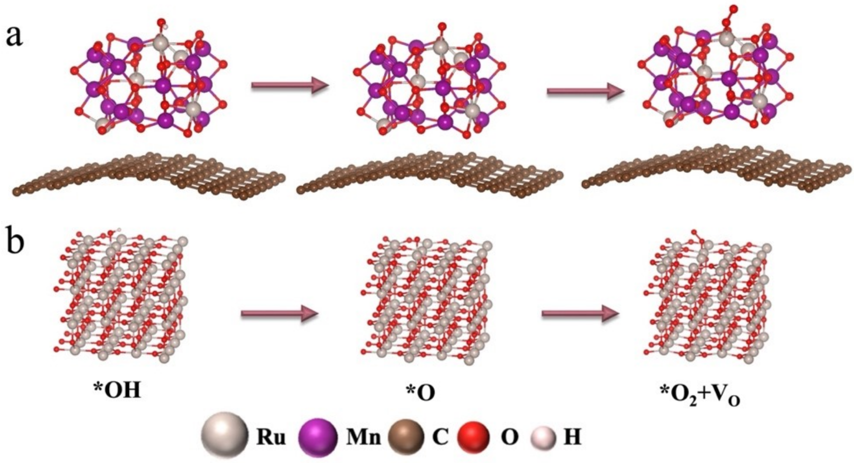

FigureS20 Structures of key intermediates for (a) CNT-($Mn_{0.75}Ru_{0.25}$)O_2 and (b) RuO_2(110) in the LOM reaction pathways.

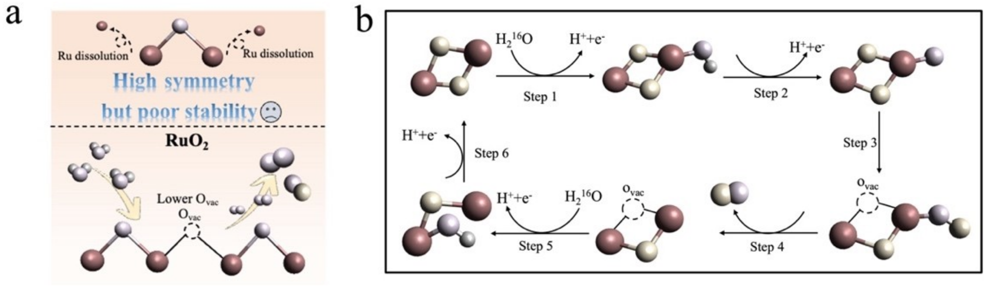

Figure S21 Schematic illustration of the LOM pathway during acidic OER. (a-b) Conventional LOM process on RuO_2

Table S1. Peak fitting results for Mn 2p in XPS analysis.

	Mn 2p					
	$2p_{3/2}$			$2p_{1/2}$		
	4+	3+	SAT	4+	3+	SAT
MnO_2	642.42	/	645.69	653.79	/	656.9
CNT-MnO_2	642.04	640.8	643.93	653.65	652.4	655.57
CNT-$(Mn_{0.75}Ru_{0.25})O_2$	642.28	641.05	644.55	653.78	652.599	656.3

Table S2. Peak fitting results for Ru 3p in XPS analysis.

Ru 3p				
	$2p_{3/2}$		$2p_{1/2}$	
	4+	3+	4+	3+
RuO_2	462.54	465.64	484.89	487.89
$(Mn_{0.75}Ru_{0.25})O_2$	462.15	465.21	484.44	487.37
CNT-$(Mn_{0.75}Ru_{0.25})O_2$	461.91	465.05	484.34	487.03

Table S3. Structure parameters extracted from the EXAFS fitting of Ru K-edge

Sample	Path	*CN*[a]	R (Å)[b]	σ^2 c(Å^2)[c]	R factor[d]
RuO_2	Ru-O	4.576	1.96	0.005	0.02
CNT-($Mn_{0.75}Ru_{0.25}$)O_2	Ru-O	2.938	1.95	0.002	0.01
	Ru-Ru/Mn	0.324	2.76	0.005	

[a]*CN*: coordination numbers; [b]R: bond distance; [c]σ^2: Debye-waller factors; [d]R factor: goodness of fit. According to the experimental EXAFS fit of Ru foil and RuO_2 references by fixing *CN* as known crystallographic value, the S0 was both set to 1.0.

Table S4. The mass percentage of Ru in CNT-($Mn_{0.9}Ru_{0.1}$)O_2, CNT-($Mn_{0.8}Ru_{0.2}$)O_2, CNT-($Mn_{0.75}Ru_{0.25}$)O_2 and CNT-($Mn_{0.7}Ru_{0.3}$)O_2 based on the ICP-OES results.

	**CNT-($Mn_{0.9}Ru_{0.1}$)O_2**	**CNT-($Mn_{0.8}Ru_{0.2}$)O_2**	**CNT-($Mn_{0.75}Ru_{0.25}$)O_2**	**CNT-($Mn_{0.7}Ru_{0.3}$)O_2**
Ru (wt. %)	1.75	3.15	3.85	4.88

Table S5. Summary of OER catalytic performance of representative noble-metal-based electrocatalysts in acidic media.

Catalysts	Electrolyte	η_{10} (mV)	Mass activity @η (A g^{-1}_{Ru} @mV)	Stability@ Current density (h@mA cm^{-2})	References
CNT-($Mn_{0.75}$Ru0$_{.25}$)O_2	0.1 M $HClO_4$	120	5545@270	160@10	This work
NC@Vo·-RuO_2/CNTs-350	0.1 M $HClO_4$	170	1738@250	900@10	Ref.[6]
β-MnO_2-Ru	0.1 M $HClO_4$	278	2144@300	50@10	Ref.[7]
$Ru_1Ir_1O_x$	0.5 M H_2SO_4	204	1124@300	100@10	Ref.[8]
$Mn_{0.73}Ru_{0.27}O_{2-\delta}$	0.5 M H_2SO_4	208	879@270	10@10	Ref.[9]
$Ir_{0.06}Co_{2.94}O_4$	0.1 M $HClO_4$	292	2511@300	200@10	Ref.[10]
5.1%$IrMnO_2$	0.5 M H_2SO_4	239	191.8@300	1600@100	Ref.[11]
$Mn_{0.2}RuO_2$	0.5 M H_2SO_4	188	115.2@210	150@10	Ref.[12]
Ir/MnO_X	0.1 M $HClO_4$	200	4000@300	200@10	Ref.[13]
$RuCoO_x$	0.1 M $HClO_4$	200	2278@250	100@10	Ref.[14]
m-$Ir_xRu_{1-x}O_2$	0.1 M $HClO_4$	180	558.13@270	256@900	Ref.[15]
RuO_2/MnO_2/CC	0.5 M H_2SO_4	189	229.67@250	800@10	Ref.[16]
12Ru/MnO_2	0.1 M $HClO_4$	160	1264@165	200@10	Ref.[17]

Table S6. Comparison of OER activity and Ru loading of CNT-$(Mn_{0.75}Ru_{0.25})O_2$ with representative Ru-based catalysts in acidic media

Catalyst	Ru (wt.%)	Mass loading of Ru (mg cm^{-2})	η_{10} (mV)	Mass activity @η (A g^{-1}_{Ru} @mV)	References
CNT-$(Mn_{0.75}Ru_{0.25})O_2$	3.85	0.077	120	5545@270	This work
$RuMnO_x$@RuO_x−1.5	7.02	0.09	192	1645@270	Ref.[18]
$RuTiO_x$	92.3	0.186	198	403@270	Ref.[19]
BCN-0.5Ru	6.01	0.006	164	/	Ref.[20]
Co-Ru@RuO_2	90.5	0.45	203	200@270	Ref.[21]
$RuCoO_x$	5.0	0.043	200	2278@250	Ref.[22]
RuCo	94.5	0.945	210	/	Ref.[23]
$MnRuO_x$-300	15.01	0.03	231	/	Ref.[24]
$Ru_{0.48}Mn_{0.52}O_2$	16.3	0.114	154	/	Ref.[25]
$Mn_{0.75}Ru_{0.25}O_2$	42.07	0.1	154	/	Ref.[26]
NC@Vo·-RuO_2/CNTs-350	32.27	0.085	170	1738@250	Ref.[6]
12Ru/MnO_2	11.6	0.232	161	1264@165	Ref.[27]
(Ce W)-RuO_2	/	0.080	206	966@250	Ref.[28]

Rreference

1 Hohenberg, P. & Kohn, W. Inhomogeneous Electron Gas. Physical Review 136, B864-B871

2 W, Kohn. & L. J, Sham,. Physical Review 1965 **140**, A1133-A1138,

3 G, Kresse. & J,Furthmüller Physical review B 199654, 11169

4 P. E, Blöchl. Projector. Physical Review B **50**, 17953-17979

5 J. P, Perdew. K Burke. & M, Ernzerhof Physical Review Letters 1996 77, 3865.

6 H. Yan, Z. Jiang, B. Deng, Y. Wang and Z.-J. Jiang, *Adv.Energy Mater.*, 2023, **13**, 2300152.

7 Y. Qin, Y. Liu, Y. Zhang, Y. Gu, Y. Lian, Y. Su, J. Hu, X. Zhao, Y. Peng, K. Feng, J. Zhong, M. H. Rummeli and Z. Deng, *ACS Catal.*, 2023, **13**, 256–266.

8 J. He, X. Zhou, P. Xu and J. Sun, *Adv.Energy Mater.s*, 2021, **11**, 2102883.

9 K. Wang, Y. Wang, B. Yang, Z. Li, X. Qin, Q. Zhang, L. Lei, M. Qiu, G. Wu and Y. Hou, *Energy Environ. Sci.*, 2022, **15**, 2356–2365.

10J. Shan, C. Ye, S. Chen, T. Sun, Y. Jiao, L. Liu, C. Zhu, L. Song, Y. Han, M. Jaroniec, Y. Zhu, Y. Zheng and S.-Z. Qiao, *J. Am. Chem. Soc.*, 2021, **143**, 5201–5211.

11S. Wu, Z. Chen, Z. Qian, Z. Zhang, Y. Sun, X. Zhang, H. Tao, Q. Zhang, S. Xie and Y. Wang, *Adv.Funct.Mater.*, 2417766.

12 Q. Ji, B. Tang, X. Zhang, C. Wang, H. Tan, J. Zhao, R. Liu, M. Sun, H. Liu, C. Jiang, J. Zeng, X. Cai and W. Yan, *Nat Commun.*, 2024, **15**, 8089.

13 D. Wang, F. Lin, H. Luo, J. Zhou, W. Zhang, L. Li, Y. Wei, Q. Zhang, L. Gu, Y. Wang, M. Luo, F. Lv and S. Guo, *Nat Commun*, 2025, **16**, 181.

14 W. Zhu, F. Yao, K. Cheng, M. Zhao, C.-J. Yang, C.-L. Dong, Q. Hong, Q. Jiang, Z. Wang and H. Liang, *J. Am. Chem. Soc.*, 2023, **145**, 17995–18006.

15 K. Qin, H. Yu, W. Zhu, Y. Zhou, Z. Guo, Q. Shao, Y. Wu, X. Wang, Y. Li, Y. Ji, F. Liao, Y. Liu, Z. Kang and M. Shao, *Adv. Funct. Mater.*, 2024, 2402226.

16 W. Jia, X. Cao, X. Chen, H. Qin, L. Miao, Q. Wang and L. Jiao, *Small*, 2024, 2310464.

17 C. Lin, J.-L. Li, X. Li, S. Yang, W. Luo, Y. Zhang, S.-H. Kim, D.-H. Kim, S. S. Shinde, Y.-F. Li, Z.-P. Liu, Z. Jiang and J.-H. Lee, *Nat Catal*, 2021, **4**, 1012–1023.

18Y. Qin, X. Niu, R. Zhao, J. Sun, Z. Xu, Z. Guo, D. Liu, L. Guo, C. Liu, J. Zhang and Q. Wang, *ACS Catal.*, 2024, **14,** 12970−12981

19W. Hu, B. Huang, M. Sun, J. Du, Y. Hai, W. Yin, X. Wang, W. Gao, C. Zhao, Y. Yue, Z. Li and C. Li, *Advanced Materials*, 2025, **37**, 2411709.

20X. Bai, X. Zhang, Y. Sun, M. Huang, J. Fan, S. Xu and H. Li, *Angew. Chem. Int. Ed.*, 2023, **135**, e202308704.

21J. Chen, Y. Ma, C. Cheng, T. Huang, R. Luo, J. Xu, X. Wang, T. Jiang, H. Liu, S. Liu, T. Huang, L. Zhang and W. Chen, *J. Am. Chem. Soc.*, 2025, **147**, 8720–8731.

22 W. Zhu, F. Yao, K. Cheng, M. Zhao, C.-J. Yang, C.-L. Dong, Q. Hong, Q. Jiang, Z. Wang and H. Liang, *J. Am. Chem. Soc.*, 2023, **145**, 17995–18006.
23 Z. Jiang, L. Shao, Y. Sun, Y. Dong, X. Zheng, T. Wang, J. Li, H. Shao, L. Jiao and Y. Deng, *Adv. Funct. Mater.*, e09656.
24 J. Zhang, L. Xu, X. Yang, S. Guo, Y. Zhang, Y. Zhao, G. Wu and G. Li, *Angew. Chem. Int. Ed.*, ,2024, e202405641.
25 Z. Li, H. Sheng, Y. Lin, H. Hu, H. Sun, Y. Dong, X. Chen, L. Wei, Z. Tian, Q. Chen, J. Su and L. Chen, *Adv. Funct. Mater.*, 2024, 2409714.
26 D. Zhou, Y. Chang, J. Tang and P. Ou, *Small*, 2024, 2412265.
27 H. Liu, Q. Zhou, J. Yu, M. Nakabayashi, Y.-T. Lee, N. Shibata, Y. Li and J.-J. Delaunay, *ACS Catal.*, 2025, **15**, 8511–8521.
